# Optical and pharmacological manipulation of hypoglossal motor nucleus identifies differential effects of taltirelin on sleeping tonic motor activity and responsiveness

**DOI:** 10.1038/s41598-023-39562-z

**Published:** 2023-07-29

**Authors:** Jasmin Aggarwal, Raina Ladha, Wen-Ying Liu, Hattie Liu, Richard L. Horner

**Affiliations:** 1 Department of Physiology, University of Toronto, 3206 Medical Sciences Building, 1 King’s College Circle, Toronto, ON M5S 1A8 Canada; 2 Department of Medicine, University of Toronto, 3206 Medical Sciences Building, 1 King’s College Circle, Toronto, ON M5S 1A8 Canada

**Keywords:** Respiration, Sleep

## Abstract

Pharyngeal muscle activity and responsiveness are key pathophysiological traits in human obstructive sleep apnea (OSA) and strong contributors to improvements with pharmacotherapy. The thyrotropin-releasing hormone (TRH) analog taltirelin is of high pre-clinical interest given its neuronal-stimulant properties, minimal endocrine activity, tongue muscle activation following microperfusion into the hypoglossal motor nucleus (HMN) or systemic delivery, and high TRH receptor expression at the HMN compared to rest of the brain. Here we test the hypothesis that taltirelin increases HMN activity and/or responsivity to excitatory stimuli applied across sleep–wake states in-vivo. To target hypoglossal motoneurons with simultaneous pharmacological and optical stimuli we used customized “opto-dialysis” probes and chronically implanted them in mice expressing a light sensitive cation channel exclusively on cholinergic neurons (ChAT–ChR2, n = 12) and wild-type mice lacking the opsin (n = 10). Both optical stimuli applied across a range of powers (*P* < 0.001) and microperfusion of taltirelin into the HMN (*P* < 0.020) increased tongue motor activity in sleeping ChAT–ChR2 mice. Notably, taltirelin increased tonic background tongue motor activity (*P* < 0.001) but not responsivity to excitatory optical stimuli across sleep–wake states (*P* > 0.098). This differential effect on tonic motor activity versus responsivity informs human studies of the potential beneficial effects of taltirelin on pharyngeal motor control and OSA pharmacotherapy.

## Introduction

Obstructive sleep apnea (OSA) is a common and widespread disorder with serious clinical, social, and economic burdens^1,2^. There are identifiable pathophysiological traits (‘*endotypes*’) that by themselves and collectively predispose an individual to OSA^3–6^. The end-result in all cases, however, is that the upper airway closes only in sleep and not in wakefulness. A key component of OSA pathogenesis is the impact of sleep mechanisms on pharyngeal muscle tone and compensatory motor responses. Identifying these mechanisms is a prerequisite to identifying rational targets and strategies to manipulate those mechanisms to benefit patients^7–10^.

Findings in animal models identified that an endogenous noradrenergic drive contributes a tonic excitation to the hypoglossal motor nucleus that is withdrawn in sleep^11–15^. Findings also identify that a muscarinic receptor mechanism mediates the strong hypoglossal motor inhibition in rapid eye movement (REM) sleep^16^. Those findings from animal models constituted the first identification of mechanisms controlling output to the major pharyngeal muscle in sleep that is central to OSA in humans, and led to selection of cholinergic-noradrenergic drug combinations for potential OSA pharmacotherapy^8,17–20^.

An un-biased screen identified additional targets of high interest to modulate hypoglossal activity for potential OSA pharmacotherapy, and provided a database of them with FDA-approved drugs^10^. The muscarinic and noradrenergic receptors that are major controllers of tongue motor activity in sleep^12,16^ and the successful targets in OSA pharmacotherapy^8,17–20^ were identified from that screen^10^. The screen was also the reason that taltirelin, a thyrotropin-releasing hormone (TRH) analog, was the focus of recent investigations to identify effects on pharyngeal muscle activity^21,22^. TRH receptor RNA shows a high degree of differential expression at the hypoglossal motoneuron pool, being 6.3‐fold higher compared to the rest of the brain^10^, with strong projections of TRH-positive neurons to the hypoglossal motor nucleus^23–25^.

Taltirelin has minimal endocrine activity but favorable neural stimulant properties, including selectively increasing tongue motor activity in sleep with either microperfusion into the hypoglossal motor nucleus in rats or systemic delivery^21^. However, although a change in prevailing tongue muscle activity is a useful and convenient measure of the activating response to pharmacological interventions at the hypoglossal motor nucleus, e.g., as in previous studies^12,16,21,22^, it does not capture another key property of pharyngeal motor control that is particularly relevant to OSA pathogenesis and pharmacotherapy. Pharmacotherapy can be effective in reducing OSA severity via increasing pharyngeal muscle activity and/or motor responsiveness.

Pharyngeal muscle responsiveness is one of the key pathophysiological traits in human OSA and a strong contributor to improvements in OSA severity with pharmacotherapy^7,8,17–19^. In human studies, for example, this measure is captured from the pharyngeal muscle response to increasing excitatory drive during apnea and can increase over threefold with pharmacotherapy^17^. We developed tools to characterize a parallel measure in mice. Using acute and temporally precise optical control of genetically targeted cells, i.e., ‘optogenetics’^26^, we identify hypoglossal motor responsiveness from the activation response to discrete excitatory stimuli applied across sleep–wake states^27^. In the present study, we combine simultaneous microperfusion of the hypoglossal motor nucleus with optical stimulation of hypoglossal motoneurons in genetically modified mice that express channelrhodopsin-2 (ChR2) on cholinergic neurons to identify effects of the TRH analog taltirelin on hypoglossal motor activity and responsiveness across sleep–wake states in-vivo.

## Results

### Sites of opto-dialysis

**Figure 1:**
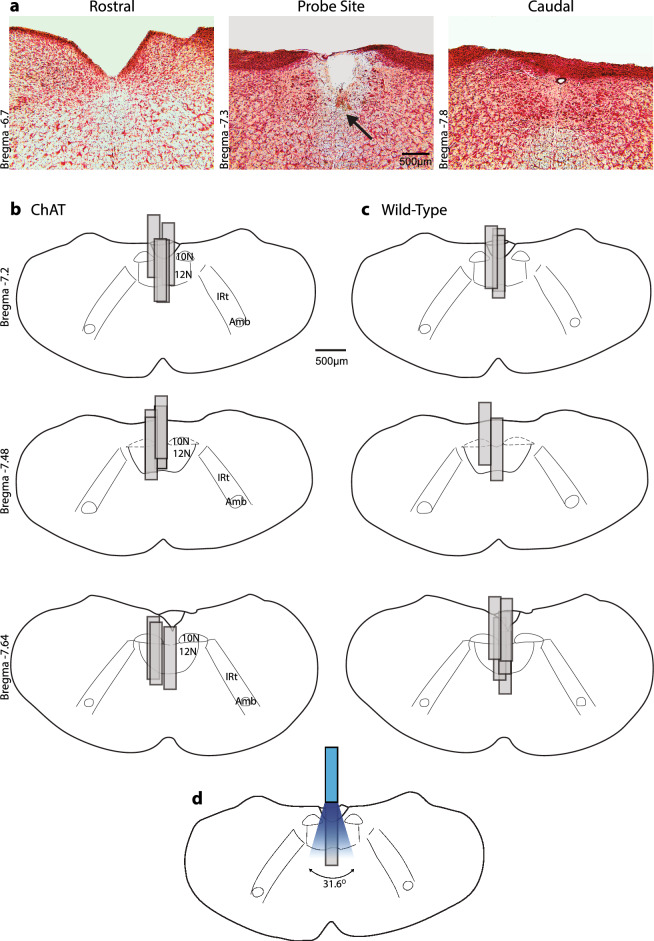
Example (**a**) and group (**b**,**c**) data showing the sites of microdialysis on coronal diagrams of the mouse medulla from all the experiments in the ChAT–ChR2 and wild-type mice. The ventral tips of the probe sites are identified (see arrow, **a**), and sites of microdialysis reconstructed and plotted to scale on representative standard sections (**b**,**c**); overlap obscures some of the individual dialysis sites. The sites of microdialysis perfusion were located within or immediately adjacent to the hypoglossal motor nucleus in all experiments. The tip of the microdialysis probe extended 1 mm farther than the tip of the optical fiber. An example is shown (**d**) of a site of optical stimulation with the calculated divergence angle (see “Methods” section) of the light stimulation from the tip of the optical fiber. The diminution of power with increasing distance from the fiber tip is represented as decreased intensity of the blue light for visual purposes only. *Abbreviations:* 12N, hypoglossal motor nucleus; 10N, dorsal motor nucleus of the vagus; IRt, intermediate medullary reticular region; Amb, nucleus ambiguous.

Figure 1 shows an example of a microdialysis probe site in the hypoglossal motor nucleus, and the distribution of probe sites from each experiment in each of the ChAT–ChR2 mice and the wild-type mice lacking the opsin. The sites of microdialysis perfusion were located within or immediately adjacent to the hypoglossal motoneuron pool in all experiments. Given that the tip of the microdialysis probe extended 1 mm farther than the tip of the optical fiber (see “Methods” section, so that the active portion of the dialysis membrane was within the area of the light emitted from the optical fiber), then the tip of the optical probe was at or just above the surface of the medulla at a position equivalent to the dorsal tip of the microdialysis probe membrane (Fig. 1d). Figure 1 also shows an example of the calculated divergence angle of the light stimulation from the tip of the optical fiber (see “Methods” section).

### Motor activity and responses to optical stimuli and taltirelin

**Figure 2:**
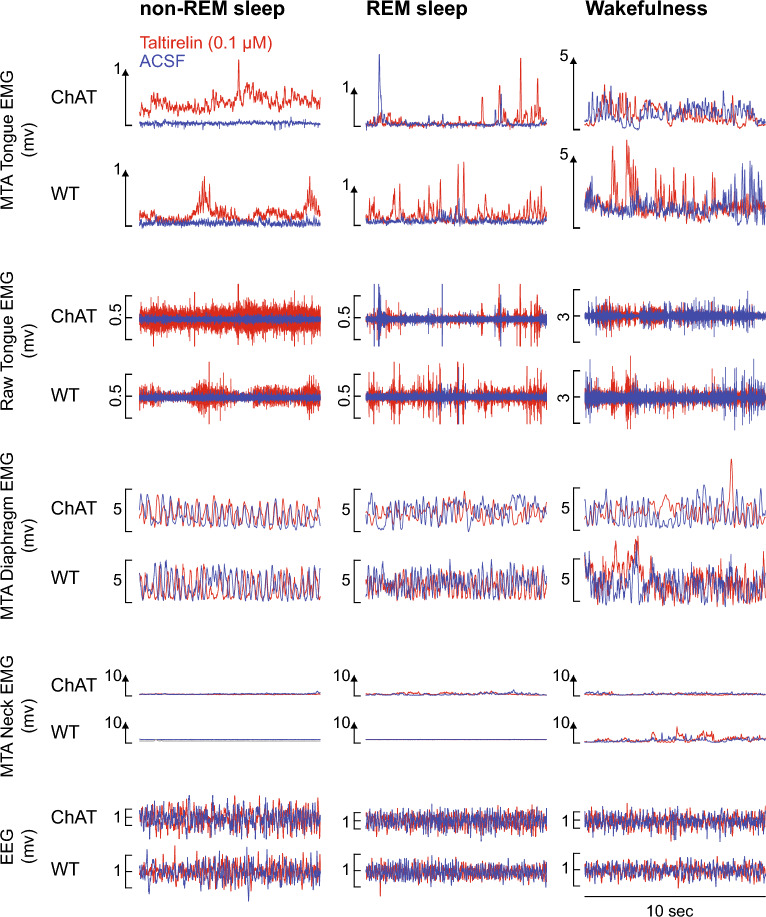
Representative traces of baseline tongue muscle activity (i.e., in the absence of optical stimulation) recorded from one individual wild-type (WT) mouse and one ChAT mouse across sleep–wake states with and without microperfusion of taltirelin into the hypoglossal motor nucleus. Note the increased tongue motor activity in the presence of taltirelin compared to artificial cerebrospinal fluid (ACSF) in the ChAT and WT mice. The pairs of traces in each case are superimposed to show the augmentation of tongue motor activity by taltirelin, and similarity in other signals such as diaphragm and neck electromyogram (EMG) activities and the electroencephalogram (EEG). *Abbreviations:* MTA, moving time average.

Figure 2 shows representative baseline tongue muscle activity (i.e., in the absence of optical stimulation) recorded across sleep–wake states with and without microperfusion of taltirelin into the hypoglossal motor nucleus. Tongue muscle activity with ACSF was minimal and tonic (i.e., without within-breath phasic respiratory-related activity) in both non-REM and REM sleep, except for periods of transient tongue muscle activations that accompany and typify REM sleep. Activity in wakefulness was also associated with transient phasic activations that are typical of freely behaving mice (Fig. 2). Also note the increased tongue motor activity in the presence of taltirelin at the hypoglossal motor nucleus compared to ACSF in the ChAT and wild-type mice.

**Figure 3:**
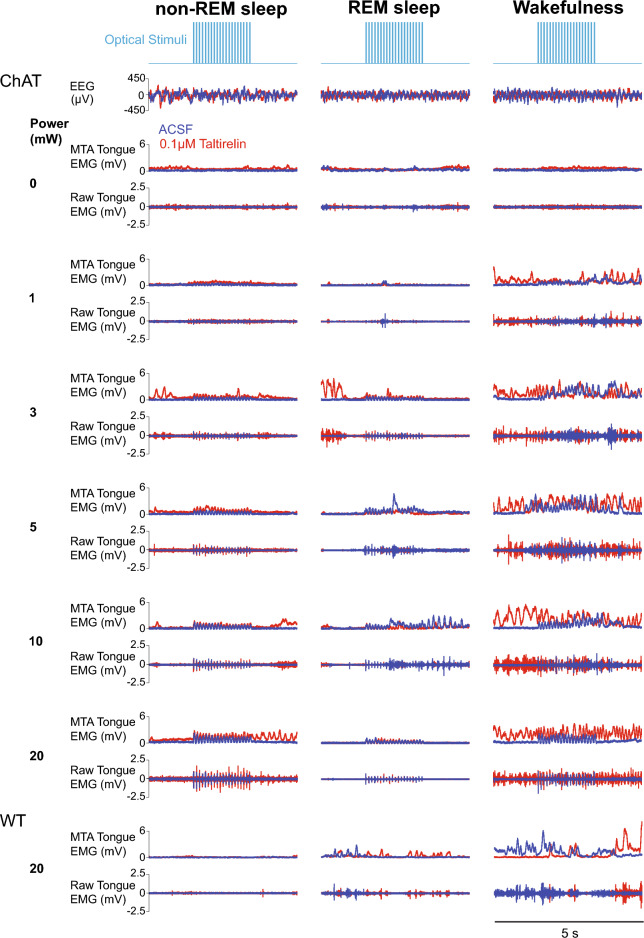
Examples of tongue motor responses in ChAT–ChR2 mice elicited by optical stimulation in sample periods of wakefulness, non-REM sleep and REM sleep, with and without microperfusion of taltirelin into the hypoglossal motor nucleus. Typically, in each sleep and awake state the magnitude of the elicited tongue motor responses increased with increasing power of stimulation, with lesser responses in REM sleep compared to the other states. Baseline tonic tongue motor activity also increased in the presence of taltirelin, but motor responses to the excitatory pulses of optical stimuli were typically unchanged with taltirelin versus ACSF. No tongue motor responses were observed with optical stimulation in the wild-type (WT) mice lacking the opsin. Traces are from one individual WT mouse and one ChAT mouse. *Abbreviations:* EEG, electroencephalogram; EMG, electromyogram; MTA, moving time average.

Figure 3 shows examples of the tongue motor responses in ChAT–ChR2 mice elicited by optical stimulation in sample periods of wakefulness, non-REM sleep and REM sleep, with and without microperfusion of taltirelin into the hypoglossal motor nucleus. Note that: (i) within any given sleep–wake state, the magnitude of the elicited tongue motor responses increased with increasing power of optical stimulation, (ii) tongue motor responses to optical stimulation in REM sleep were typically reduced compared to waking and non-REM sleep, and (iii) baseline tonic tongue motor activity increased in the presence of taltirelin, but motor responses to the excitatory pulses of optical stimuli were typically unchanged with taltirelin versus ACSF. No tongue motor responses were observed with optical stimulation in the wild-type mice lacking the opsin.

The data set was 99.4% complete for interventions applied across all mice at all applied powers (i.e., 0, 1, 3, 5 10 and 20 mW) across wakefulness, non-REM sleep and REM sleep with microperfusion of ACSF and 0.1 μM taltirelin into the hypoglossal motor nucleus. The only exceptions were: (i) a total of two missing values in the group of wild-type mice (one mouse in wakefulness with ACSF at 0 mW, and one in REM sleep with 0.1 μM taltirelin at 1 mW), and (ii) a total of three missing values in the group of ChAT–ChR2 mice (two mice only with ACSF in REM sleep at 3 mW, and one with 0.1 μM taltirelin in REM sleep at 1 mW). Data were also obtained in wakefulness with microperfusion of 1 μM taltirelin into the hypoglossal motor nucleus but no data were obtained in sleep with 1 μM taltirelin as the mice remained awake.

**Figure 4:**
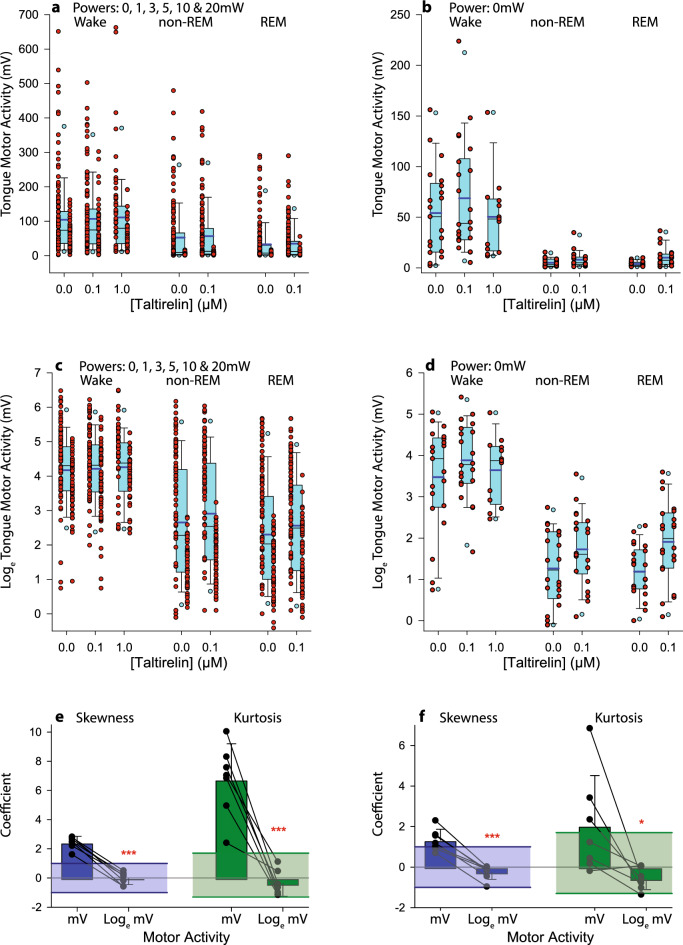
Distribution of the magnitude of tongue motor responses to optical stimulation of the hypoglossal motor nucleus. The box and whisker plots (blue boxes in panels **a** to **d**) show the group data as median (solid line), mean (thicker solid line), 25th and 75th percentiles, and data points indicating the 5th and 95th percentiles. The individual data points superimposed on each plot (red symbols) show average responses to optical stimulation at each indicated power at each concentration of taltirelin at the hypoglossal motoneuron pool for each of wakefulness, non-REM and REM sleep. Each response in each mouse is indicated by a discrete symbol (n = 12 ChAT, data shown on the left of each boxplot, and n = 10 wild-type, data shown on the right of each boxplot). The natural logarithm (log_e_) of the motor responses normalized the distributions such that the coefficients of skewness and kurtosis of the data were significantly reduced to within the range for normal distribution (skewness: <  ± 1, blue solid line and blue box; kurtosis: 1.7 to − 1.3, green solid lines and green box, panels a and f). *Abbreviations:* ***P < 0.001; *P < 0.05.

Figure 4 shows the distribution of the magnitude of tongue motor responses to optical stimulation of the hypoglossal motor nucleus across all powers as well as baseline motor activity without optical stimulation (i.e., 0 mW). Analysis showed that the data were not normally distributed with positive skew (Fig. 4a,b). Taking the natural logarithm (log_e_) of the motor responses normalized the distribution (Fig. 4c,d). Analysis (Fig. 4e,f) confirmed that taking the log_e_ of the motor responses significantly reduced the coefficients of skewness (t_6_ > 9.43, *P* < 0.001) and kurtosis (t_6_ > 2.72, *P* < 0.035, paired t-tests). Moreover, the coefficients of skewness and kurtosis of the data set after normalization were reduced to within the range considered to be normally distributed (skewness: <  ± 1 and kurtosis: 1.7 to − 1.3^28^, Fig. 4e,f). Parametric statistical analyses were therefore performed on the log_e_ transformed data.

**Figure 5:**
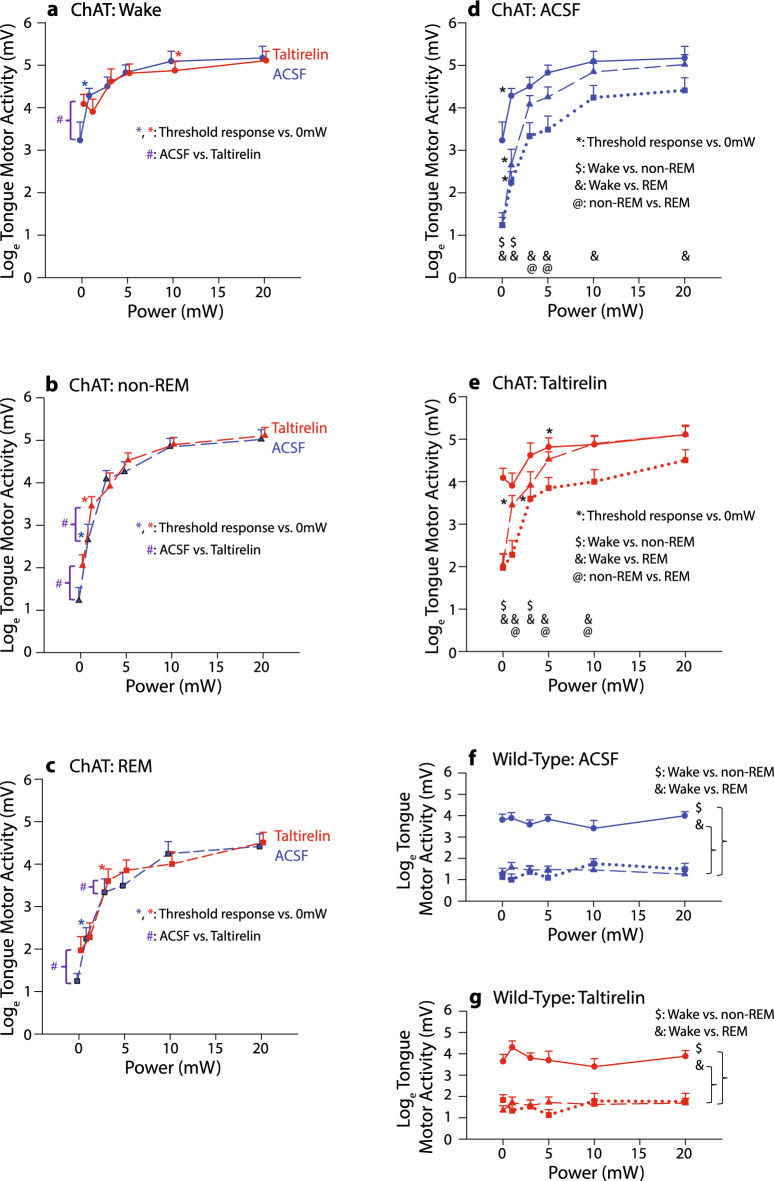
Group mean (+ SEM) data showing the effect of optical stimulation power (0, 1, 3, 5, 10 and 20 mW) on tongue motor responses in wakefulness, non-REM and REM sleep with microperfusion of taltirelin or vehicle (i.e., artificial cerebrospinal fluid, ACSF) into the hypoglossal motor nucleus in ChAT–ChR2 and wild-type mice. Statistically significant differences are indicated by the respective symbols in panels (**a**) to (**g**): *threshold response at the indicated power versus 0 mW (i.e., sham) controls; #, ACSF versus taltirelin for the indicated comparisons; $, wakefulness versus non-REM sleep; &, wakefulness versus REM sleep, and @, non-REM versus REM sleep. See text for further details.

Figure 5 shows group data for the tongue motor responses to optical stimulation of the hypoglossal motor nucleus (i.e., 0, 1, 3, 5 10 and 20 mW) across sleep–wake states with and without microperfusion of taltirelin. Overall, the data in Fig. 5 as well as Figs. 2 and 3 show that taltirelin at the hypoglossal motor nucleus increased tonic background tongue motor activity but motor responses to the pulses of excitatory optical stimuli (responsivity) were unchanged in the presence of taltirelin.

In wakefulness, non-REM sleep and REM sleep there was a statistically significant main effect of optical stimulation power on tongue motor responses in the ChAT–ChR2 mice (Fig. 5a–c, each F_5,55_ > 13.24, each *P* < 0.001, 2-way ANOVA-RM), i.e., increased motor responses were elicited by increased power of optical stimulation. Likewise, there was a statistically significant main effect of taltirelin at the hypoglossal motor nucleus in stimulating tongue motor activity in non-REM and REM sleep compared to the ACSF vehicle controls (F_1,11_ > 7.47, *P* < 0.020, 2-way ANOVA-RM, post-hoc paired t-tests < 0.020), but not in wakefulness likely due to varying background activity related to waking behaviors (F_1,11_ = 0.36, *P* = 0.561).

Analyses also showed that the level of tongue motor responses to optical stimulation depended on the presence of taltirelin at the hypoglossal motor nucleus in each of wakefulness, non-REM, and REM sleep, i.e., there was a statistically significant interaction (each F_5,55_ > 2.51, each *P* < 0.042, range of *P* = 0.001 to 0.041, 2-way ANOVA-RM, Fig. 5a–c). Post-hoc analyses identified that tongue motor activity at the time of the optical stimuli was significantly increased by taltirelin, compared to the ACSF vehicle controls, only with the sham controls (0mW stimuli) for each of wakefulness, non-REM sleep and REM sleep (each t_11_ > 3.34, *P* ≤ 0.001, post-hoc paired-t-tests, see symbols ‘#’ at 0mW in Fig. 5a–c): i.e., taltirelin increased the prevailing level of tonic background tongue motor activity, a finding consistent with Figs. 2 and 3. However, the peak optically-elicited tongue motor responses above 0mW were unaffected by the presence of taltirelin at the hypoglossal motor nucleus, i.e., the responses were not significantly different from the optically-elicited motor responses during the ACSF vehicle controls across the range from 1-20mW (range of *P* = 0.099 to 0.958 from post-hoc paired-t-tests, except for two comparisons: 1mW in non-REM sleep and 3mW in REM sleep as indicated by the additional symbols ‘#’ Fig. 5a–c).

The group data also showed a statistically significant effect of sleep–wake state on tongue motor activity in both the ChAT–ChR2 mice and the wild-type mice lacking the opsin (Fig. 5d–g). In the ChAT–ChR2 mice there was a significant activating effect of stimulation power on peak tongue motor responses that depended on sleep–wake state with both ACSF and taltirelin at the hypoglossal motor nucleus (each *P* < 0.001, 2-way ANOVA-RM, Fig. 5d,e). Motor responses were typically and significantly reduced in REM sleep compared to wakefulness across the range of stimulation powers (*P* < 0.05, symbols ‘&’ in Fig. 5d,e) and reduced from wakefulness to non-REM sleep and from non-REM to REM sleep at some powers (*P* < 0.05, symbols ‘$’ and ‘@’ in Fig. 5d,e). As expected, there was no response to optical stimulation in the wild-type mice lacking the opsin with either ACSF or taltirelin (each F_5,45_ < 0.77, each *P* > 0.581, 2-way ANOVA-RM, Fig. 5f,g), and greater tongue motor activity in wakefulness compared to non-REM and REM sleep (symbols ‘$’ and ‘&’ in Fig. 5d,e).

In the ChAT–ChR2 mice and wild-type mice there was a significant main activating effect of taltirelin on tongue motor activity, and a significant effect of sleep–wake states (both *P* < 0.001, 2-way ANOVA-RM). The activating effect of taltirelin on tongue motor activity depended on sleep–wake state (*P* = 0.393, 2-way ANOVA-RM) with tongue motor activity during ACSF and taltirelin being significantly larger in wakefulness compared to non-REM and REM sleep (all *P* < 0.001, post-hoc paired t-tests).

## Discussion

Here we targeted the hypoglossal motor nucleus with simultaneous pharmacological and optical stimuli using customized “opto-dialysis” probes in mice expressing a light sensitive cation channel exclusively on cholinergic neurons^27,29^. This approach provides for (i) acute and temporally precise optical control of cholinergic hypoglossal motoneurons^26,29^, (ii) in combination with their targeted manipulation with a pharmacological agents of translational interest, (iii) while also recording the immediate direct output of those motoneurons as tongue motor activity. Taltirelin was chosen as the pharmacological agent given its action as an upper airway-preferring respiratory stimulant and potential for OSA pharmacotherapy^21,22,30^. Taltirelin is also of interest as a potential reversal agent for opioid-induced respiratory depression^31,32^. ‘Optogenetics’ was chosen as the tool to interrogate hypoglossal motor circuitry given the capacity to quantify pharyngeal muscle motor responsiveness across sleep–wake states^27^. Pharyngeal muscle responsiveness is one of the key pathophysiological traits in human OSA and the strongest contributor to improvements in OSA severity with pharmacotherapy^7,8,17–19^.

Here we identified that optical stimuli applied across a range of powers and microperfusion of taltirelin into the hypoglossal motor nucleus increased tongue motor activity in sleep in ChAT–ChR2 mice. Notably, however, although taltirelin increased the prevailing level of tonic background tongue motor activity it did not alter the motor responses to excitatory optical stimuli applied across sleep–wake states (Fig. 5). This differential effect of taltirelin on baseline hypoglossal motor activity versus responsivity identifies properties of hypoglossal motoneurons that would not (or could not) otherwise be identified in-vivo.

Taltirelin was chosen for these ‘opto-dialysis’ studies to identify its effects on upper airway motor tone and responses to discrete excitatory stimuli provided by optical stimulation. This choice followed our recent identification of the properties and responses of taltirelin compared to TRH^21,22^. TRH at the hypoglossal motor nucleus activates tongue motor activity but the effects are biphasic and short-lasting (over minutes) whereas the effects of the TRH analog taltirelin are sustained and maintained (over hours). Taltirelin is also of high pre-clinical interest given identification of increased tongue muscle activity in sleep with microperfusion into the hypoglossal motor nucleus or systemic delivery^21,22^. Taltirelin’s receptor target shows high (6.3-fold) differential expression at the hypoglossal motor nucleus compared to the rest of the brain^10^ consistent with strong TRH-positive neuronal projections to the hypoglossal motoneuron pool and TRH receptors on hypoglossal motoneurons^23–25^.

The concentrations of taltirelin used in the present study were chosen and constrained by the potential for arousal responses elicited by their brainstem application. Microperfusion of 1–10 μM taltirelin into the hypoglossal motoneuron pool in rats, for example, can lead to increased percent time spent in active wakefulness and corresponding relative decreases in quiet wakefulness^21,22^. Such an arousal effect is likely elicited by ‘spillover’ of microperfused taltirelin into neighboring regions of the brainstem^33^, and is more likely in mice with their much smaller brains compared to rats. This reality probably explains why microperfusion of 1 μM taltirelin into the mouse medulla elicited wakefulness such that the optical stimuli could not be applied in sleep. In contrast, microperfusion of 0.1 μM taltirelin was sufficient for stimuli at the hypoglossal motor nucleus to be applied and analysed across wakefulness, non-REM and REM sleep. In humans, TRH administration is accompanied by breathing stimulation as well as central nervous system effects indicative of arousal such as restlessness and augmented vigilance^34^.

Taltirelin elicited effects on tonic tongue motor activity across sleep–wake states but did not change the responses to optical stimuli: i.e., taltirelin increased baseline tongue motor activity at 0mW optical stimuli (i.e., baseline control, sham) but thereafter the motor responses across the range of applied powers (1, 3, 5, 10 and 20 mW) were not statistically different with or without taltirelin (Fig. 5). The presence of tonic tongue motor activity and the absence of within-breath phasic (‘respiratory’) activity was expected based on previous studies in chronically instrumented mice such that application of tools such as ‘optogenetics’ can be used to evoke motor responses and test for changes in motor excitability^27,35^ without the attendant confounder of recruiting widespread increases in respiratory network stimulation, e.g., by using hypercapnia.

As expected, increased power of optical stimulation elicited increased tongue motor responses. Smaller neurons would be recruited preferentially with optical stimulation in any given anatomical plane, with larger neurons being recruited at higher powers and contributing to the larger resulting responses^36,37^. In addition, with increased power of optical stimulation additional neurons would be excited in deeper anatomical planes, thus also contributing to the larger responses with increased power.

There was sleep–wake state-dependency of the tongue motor responses at a given level of optical stimulation of the hypoglossal motoneuron pool, with responses in wakefulness being larger than in REM sleep and intermediate responses in non-REM. Overall, these effects are consistent with progressively decreased hypoglossal motor excitability from wakefulness to REM sleep, the ionic basis of which have been identified from intracellular recordings of hypoglossal motoneurons as due to changes in membrane polarization, input resistance and rheobase^38–40^. In this context, the potential for differential effects of taltirelin (or other potential agents) at the hypoglossal motor nucleus on baseline tongue motor activity versus responses to optical stimulation were unknown and hence the reason for study.

Regardless of sleep–wake state and the associated differences in ionic mechanisms modulating membrane potential and hypoglossal motor output, taltirelin increased baseline tongue motor activity but thereafter the motor responses across the range of optically applied powers were superimposable in each state. The cellular and ionic mechanisms responsible for this differential effect on tonic motor activity versus responsivity remain to be determined. In previous studies in anesthetized rats, both TRH and taltirelin at the hypoglossal motor nucleus increased within-breath phasic tongue muscle activity, with increased tonic activity being either not observed, transiently observed and/or only observed at higher doses^21^. Likewise, serotonin at the hypoglossal motor nucleus in anesthetized rats also preferentially activates within-breath phasic tongue muscle activity compared to tonic activity^41^. Such effects on phasic responses to depolarizing inputs in previous anesthetized studies are likely explained by increased input resistance of hypoglossal motoneurons in response to TRH or serotonin receptor activation^23,42,43^. Such an increase in input resistance would increase the magnitude of phasic hypoglossal motor output in response to a given phasic depolarizing input. Using optical stimuli to provide consistent and reproducible phasic depolarizing inputs to hypoglossal motoneurons, we identified that there was no facilitation of motor responses by taltirelin beyond the increase in tonic activity. Indeed, taltirelin only increased tonic tongue motor activity in these awaking and sleeping mice, with such a preferential effect on tonic activity also occurring in awake and sleeping rats with microperfusion of taltirelin^21^ or serotonin^44^ into the hypoglossal motoneuron pool.

To our knowledge the observed differential effect of taltirelin (or other agents) on baseline motor activity versus additional excitability has not been demonstrated previously, and is of pre-clinical interest given the potential relevance to upper airway motor control and pharmacological manipulation for clinical benefit such as OSA pharmacotherapy^10^. In this context, a certain degree of pharyngeal dilator muscle tone is necessary for adequate airflow and ventilation in an individual. In some individuals, the level of pharyngeal dilator muscle tone is below this level during sleep resulting in hypopnea or upper airway closure. In this scenario, sufficiently raising tonic and/or phasic dilator muscle tone would provide for adequate inspiratory airflow and ventilation through changes in airway size and/or stiffness. Clinically it has been identified in most OSA patients that the increase in tongue motor activity required to open the pharyngeal airway is relatively small^45^ and thus could potentially be achieved through interventions such as pharmacotherapy to raise tonic activity. Excessive motor activation, however, for example through phasic activation at arousal or through accompanying large phasic respiratory inputs may lead to ventilatory overshoot as the ventilatory response to the hypopnea is effectively amplified as the airway opens leading to subsequent instability and recurrence of apneas^3,46^. Practically, however, such an adverse effect of phasic activation being potentially destabilizing for breathing would only be expected to occur in individuals with an initial high loop gain^46^. Overall, in this scenario, a pharmacological intervention that increases baseline tonic motor activity and thus airway size and/or stiffness independent of increased responses to additional phasic depolarizing inputs may be useful, in principle, as part of the pharmacotherapy ‘toolbox’ for OSA. Whether taltirelin in clinically relevant amounts confers upper airway-preferring respiratory stimulant properties that can improve upper airway muscle activity, mechanics and/or OSA severity remains to be determined.

## Methods

Procedures followed the recommendations of the Canadian Council on Animal Care, and the Animal Care Committee of the University of Toronto approved the protocols. Mice lived on a 12 h light–dark cycle (7:00 h lights on) with ad libitum access to food and water and were housed together before surgery and individually after surgery^27^. The studies and their reporting followed the ARRIVE guidelines^47^.

As in previous experiments, studies were performed on 12 ChAT–ChR2 (H134R)-EYFP line 6 Bacterial Artificial Chromosome (BAC) transgenic male mice^27,29^ (mean ± SD body weight = 33.6 ± 4.9 g, range = 24.9 to 40.8 g). These mice express ChR2 exclusively in cholinergic neurons^27,29^ (B6; Cg-Tg(Chat-COP4*H134R/EYFP,Slc18a3)6Gfng/J; stock# 014546, The Jackson Laboratory, Bar Harbor, Maine, USA). The fusion of the opsin to enhanced yellow fluorescent protein (EYFP) allows for the visualization of ChR2–H134R expression by fluorescent microscopy^27^. The ChR2(H134R)-EYFP fusion protein is under the control of the choline acetyltransferase (ChAT) promoter and given that ChAT is responsible for acetylcholine synthesis, the ChAT gene product is exclusively expressed in cholinergic cells^27,29^. Since motoneurons are cholinergic, the hypoglossal motoneurons of ChAT–ChR2 (H134R)-EYFP mice express ChR2(H134R) and are the target of manipulation in the present studies^27^.

For the experiments in awake and sleeping ChR2(H134R)-EYFP mice, the animals were purchased individually and there was no breeding colony, a strategy designed to reduce animal numbers. Given that the mice are generated on a C57BL/6 background, control experiments were performed on 10 C57BL/6 male mice lacking the opsin (mean ± SD body weight = 33.3 ± 4.9 g, range = 27.0 to 41.0 g). In this study male mice were used to avoid the potential confounding effect of the estrus cycle on hypoglossal motor activity^48,49^, and as such the results cannot be extrapolated to females.

### ‘Opto-dialysis’ probes

To target murine hypoglossal motoneurons with simultaneous pharmacological and optical stimuli in vivo, we constructed and utilized “opto-dialysis” probes. These custom probes combined an optical fiber and microdialysis guide cannula into a single unit. To construct the opto-dialysis probes, an optical fiber with a 0.37 numerical aperture and 200 μM diameter (ThorLabs, Newton, NJ, USA) was fixed to a 4 mm microdialysis guide cannula (CXG-04, Amuza, San Diego, CA, USA) using adhesive (Lepage Speed Set Epoxy, Henkel, Mississauga, ON). The optical fiber was positioned such that the bottom segment of the fiber lay directly on top of, and parallel to, the stainless-steel shaft of the microdialysis guide. The optical fiber was positioned to extend 2 mm farther than the end of the steel shaft of the microdialysis guide. The shorter length of the guide relative to the optical fiber allowed for the opto-dialysis probe to have a narrow diameter (225 ± 5 µm) at the tip, limiting the impact of probe implantation into the brain. When a 6 mm microdialysis probe with a 1 mm dialysis membrane, 200/220 µm inside/outside diameter (CX-I-06-01, Amuza, San Diego, CA, USA) was inserted into the guide at the time of experimentation, the terminal end of the probe extended 1 mm farther than the tip of the optical fiber so that the active portion of the dialysis membrane was within the area of the light emitted from the optical fiber^27^. Overall, this design and construction allowed for simultaneous pharmacological and optical stimulation of cholinergic ChR2-expressing hypoglossal motoneurons in the genetically modified mice.

As previously described^27^, the predicted divergence angle (Θ) of the cone of illumination from the tip of the optical fiber was calculated using the following equation: Θ = sin^−1^ (NA/*n*)^50,51^; where NA is the numerical aperture of the fiber tip (0.37) and n is the refractive index of the medium^51^ which for brain tissue was taken as 1.36^50,52,53^. This value (15.8°) is considered the half-divergence angle^50^ and thus the cone of light is estimated as 31.6° with intensity diminishing with distance from the probe^54^ which is constant in each animal due to the fixed probe position.

### Anesthesia and surgical procedures for chronic implantations

Chronic implantation procedures are as previously described^27^. General anesthesia was induced by inhaled isoflurane (5%) in an induction chamber, and then maintained via a mask placed over the snout (1.5–2.5% isoflurane)^27^. Throughout surgery oxygen was administered to the inspired air (50% oxygen, balance air). Mice were also given buprenorphine (0.5 mg/kg, subcutaneous) and meloxicam (2 mg/kg, subcutaneous) for analgesia, and dexamethasone (1 mg/kg, subcutaneous) to reduce potential brain inflammation^27^. Effective anesthesia was judged by abolition of the pedal withdrawal and corneal blink reflexes^27^. Body temperature was maintained during surgery with a water pump and heating pad (T/Pump-Heat Therapy System, Gaymar, Orchard Park, NY, USA)^27^. A heating pad was also used overnight for the first post-surgery day to help the animals maintain body temperature.

The mice were implanted with electrodes to chronically record the electroencephalogram (EEG) and neck (trapezius) electromyogram (EMG) for the determination of sleep–wake states, and tongue and diaphragm electrodes for respiratory muscle recordings^27,35^. In each mouse an electrode plug containing the EEG and EMG electrodes was custom-made before surgery. Electrodes were soldered onto individual pins on a female plug (FTSH-105-03-L-D, Samtec, New Albany, IN, USA) that contained the four pairs of electrode leads and the common reference.

The leads of the diaphragm and tongue muscle recording electrodes (AS632; Cooner Wire, Chatsworth, CA, USA) were tunneled subcutaneously from a cranial incision to the site of implantation. The ends of the diaphragm and neck wires were knotted into loops that were each sutured directly onto the respective muscles using non-absorbable coated silk sutures (6-0 SP-5697G, P-10, Covidien, Dublin, Ireland). The diaphragm electrodes were sutured onto the costal diaphragm through an abdominal incision. The tongue EMG electrodes consisted of a knotted end of ~ 1 mm diameter that was buried into a pocket in the tongue muscle exposed by blunt dissection along the submentum^27,35^. The electrode pocket was then closed using a non-absorbable coated-silk suture and the electrode lead was knotted to the suture to keep the electrode tip within the pocket. To facilitate adequate electrode placements during surgery, both the tongue and diaphragm signals were monitored on loudspeaker (AM8 Audio Amplifier, Grass) to document respiratory-related activity. All the skin incisions were then closed using absorbable sutures (4-0 GL-881, CV-15, Covidien).

The mouse was then secured in a stereotaxic apparatus for implantation of the EEG electrodes and opto-dialysis probe. The bipolar EEG electrodes plus the common reference consisted of insulated stainless-steel wire (AS632; Cooner Wire, Chatsworth, CA, USA) attached to stainless steel screws (00-90xl/8 #303SS, J. I. Morris Co., MA, USA). The EEG electrodes were positioned over the left-frontal cortex (~ 1.5 mm anterior and to the left of bregma) and right parietal cortex (~ 2 mm posterior and to the right of bregma), with the reference positioned over the right frontal skull bone. One anchor screw was positioned in the left parietal skull bone^35,55^.

To prevent blockage of the microdialysis guide cannula, a 4 mm dummy cannula (CXD-04, Amuza, San Diego, CA, USA) was inserted into the guide prior to implantation. Using a dorsal approach, the opto-dialysis probe was then inserted into the brain at the midline and 7.13 ± 0.05 mm (mean ± SEM) posterior and 4.8 mm ventral to bregma. These coordinates were selected as previous experiments have shown that photo-stimulation of hypoglossal motoneurons with an optical fiber at this location in ChAT–ChR2 mice elicits robust tongue motor responses with no effects on postural or respiratory network (diaphragm) motor activities^27^. The opto-dialysis probe and electrode plug were then secured to the skull using dental cement. The cranial incision was closed around the electrode plug and optical fiber using absorbable sutures.

### Habituation

Mice were habituated to the recording environment at least nine days after surgery. To facilitate (i) insertion of the microdialysis probe into the guide cannula, (ii) connection of the electrode plug to the recording cable (NMUF 8/30-4046SJ, Cooner Wire, Chatsworth, CA, USA) and (iii) connection of the optic fiber to the optic patch cord (50/125/900-0.22, Doric Lenses, Quebec City, QC, Canada), the mice were briefly anesthetized with isoflurane in an induction chamber at least 16–18 h prior to testing (induced at 5% for ~ 30 s and maintained at 1.5–2.5% for ~ 5–10 min). This procedure was necessary to minimize the risk of any sudden mouse movement dislodging the head piece at the time of connection. The exposed tip of the optical fiber was also cleaned with 10% alcohol and the dummy cannula was removed.

Prior to insertion of the microdialysis probe into the guide cannula, the electrode plug was connected to the recording cable and recording of the EEG and EMG signals commenced. Recording the EMG signals at the time of microdialysis probe insertion also enabled preliminary confirmation of probe placement in the hypoglossal motor nucleus, as a physical disturbance of the motor pool causes a sustained increase in tongue motor activity that typically lasted less than 5 min^27,44^. Such a transient burst of tongue muscle activity was observed at the time of probe insertion in each mouse.

The recording environment consisted of a large open-topped bowl (Rodent Bowl, MD-1514, BAS) housed within an electrically shielded and soundproofed cubicle (EPC-010, BRS/LVE Inc. Laurel, MD, USA)^27^. The animals were supplied with fresh bedding, food and water, and were left free from any disturbance^27^. A video camera inside the cubicle allowed for continuous monitoring^27^.

### Recordings and protocol

The EMG signals were amplified and band-pass filtered between 100 and 1000 Hz (Super-Z head-stage amplifiers and BMA-400 amplifiers/filters, CWE Inc., Ardmore, PA, USA)^27^. The electrocardiogram was removed from the diaphragm signal using an electronic blanker (Model SB-1, CWE Inc.). The moving-time averages of the tongue, neck and diaphragm EMGs were also obtained with time constants of 50 ms, 100 ms and 100 ms respectively (Model MA-821/RSP, CWE Inc.)^56^. The signals were digitized at a sampling rate of 2000 Hz using a data acquisition system (CED 1401 and Spike version 6 software, Cambridge Electronic Design Ltd., Cambridge, UK)^27,56^. The experiments began at 0700-0830 h and were performed during the day when the mice normally sleep. Because probe insertion and connection of the recording cable and optical fiber was performed the day before the studies, mice were undisturbed on the day of the experiments.

The microdialysis probes were connected to fluorinated ethylene propylene (FEP) Teflon tubing (0.12 mm internal diameter), which was connected to 1.0 mL syringes with a zero dead space switch (Uniswitch, BAS, West Lafayette, IN, USA). Artificial cerebrospinal fluid (ACSF) was flushed through the probe continually at a flow rate of 2.0 µL/min using a syringe pump and controller (MD-1001 BAS and MD-1020 BAS). During overnight habituation the flow rate was reduced to 0.8 µL/min. The composition of ACSF was as follows: 125 mM NaCL, 3 mM KCl, 1 mM KH2PO4, 2 mM CaCl2, 1 mM MgSO4, 25 mM NaHCO3, 30 mM D-glucose. The ACSF was bubbled with CO_2_ to a pH of 7.35–7.45.

The optical fiber was connected to a laser head and power supply (LRS-0473-GFM-00050-03, Laserglow Technologies, Toronto, ON, Canada) via an optic patch cord with a numerical aperture of 0.22 and core diameter of 50 µm (50/125/900-0.22, Doric Lenses, Quebec City, QC, Canada). The larger numerical aperture and core size of the optical fiber versus the optic patch cord prevented loss of light at the connection point^57^. The laser delivered light at a wavelength of 473 nm to evoke photocurrents from the blue light sensitive ChR2^58^.

### In-vivo optical stimulation and microdialysis perfusion (‘opto-dialysis’) protocol

Signals were recorded during microdialysis of ACSF (i.e., control condition) into the hypoglossal motor nucleus. Optical stimuli of 0mW (i.e., baseline control, sham), 1, 3, 5, 10 and 20 mW (as measured from the tip of the optical fiber) were applied in random order as a block of stimuli across naturally occurring states of wakefulness, non-REM, and REM sleep. Individual optical pulses were of 10 ms duration with the total duration of the stimulus train lasting 2 s and a frequency of 10 Hz throughout. Each stimulus train was separated by at least 25 s to facilitate full recovery from potential channel desensitization^59^. Any missing powers in any given sleep–wake state were identified and applied at the end of the protocol in an attempt to obtain a full data set. An average of 69.6 stimuli (range, 42–126) were applied across states of wakefulness, non-REM and REM sleep in each mouse.

The opto-dialysis protocol was performed across each sleep–wake state with ACSF at the hypoglossal motor nucleus. Then the perfusion medium was switched to 0.1 μM taltirelin, and after at least 35 min the optical stimulus protocol was also performed across sleep–wake states. The lag time after the switch before application of stimuli was to allow for the perfusion medium containing 0.1 μM taltirelin to travel to the tip of the microdialysis probe and into the hypoglossal motor nucleus^21^. After the opto-dialysis protocol was performed with 0.1 μM taltirelin across sleep–wake states, the perfusion medium was then switched to 1 μM and repeated. These additional interventions with microperfusion of 1 μM taltirelin into the hypoglossal motor nucleus were performed in 6 ChAT–ChR2 mice and 7 wild-type mice lacking the opsin.

### Identification of sleep–wake states

Sleep–wake states were identified as previously described^27^. Arousals from sleep were identified according to the Sleep Disorders Atlas Task Force of the American Sleep Disorders Association^60^, and transitions from non-REM to REM sleep were also identified^61^. The optical stimuli were applied after at least 15–30 s of visually identified stable sleep–wake periods. After the experiments, the sleep–wake states during each applied stimulus were confirmed. Any stimuli subsequently deemed to have been applied during transitional states (e.g., drowsiness, arousal, or transitions from non-REM to REM sleep) were not included in the analyses.

### Data analyses

The raw tongue muscle signal was full wave rectified and any DC offsets removed. As in a previous study^27^, peak tongue motor activity was then measured from the rectified EMG signal from the first pulse for each repeated intervention at each power (0, 1, 3, 5, 10, and 20 mW) in wakefulness, non-REM and REM sleep during microperfusion of the hypoglossal motor nucleus with ACSF and taltirelin. All data points were measured in Spike2 using cursors and manually recorded in a spreadsheet. A grand mean for each motor response to each power in each-sleep–wake state with each perfusion medium at the hypoglossal motor nucleus was then calculated for each mouse.

### Histology

At the end of the studies the mice were overdosed with isoflurane. The rats were then perfused intracardially with 0.9% saline and 10% formalin. The brain was then removed and fixed in 10% formalin. The medullary regions were blocked and transferred to a 30% sucrose solution and subsequently cut in 40 μm coronal sections using a cryostat (CM1850, Leica, Wetzlar, Hesse, Germany). Sections were mounted and stained with neutral red. The site of the lesion left by the microdialysis probe was localized and placed on a corresponding standard cross-section using an atlas of the mouse brain^62^. The brain of one ChAT–ChR2 mouse was damaged upon processing, but since a similar burst of tongue muscle activity was also observed in that mouse when the microdialysis probe was initially inserted on the day of the experiment, the results from this mouse were included with the others for analysis.

### Statistical analysis

As previously described and analysed, each mouse served as its own control and the analyses performed for each statistical test are indicated in the text as appropriate^27^. For all comparisons, differences were considered significant if the null hypothesis was rejected at *P* < 0.05 using a two-tailed test. Where post-hoc comparisons were performed after analysis of variance with repeated measures (ANOVA-RM), a multiple comparison procedure was then used to determine significant differences^27^ (pairwise Bonferroni t-tests or Dunnett’s test versus a single control as indicated). As identified and detailed in the results, the data set was 99.4% complete. Missing values were accommodated in the ANOVA-RM by a general linear model (*Sigmaplot* version 11, Systat Software Inc., San Jose, CA, USA). Values are shown as means ± the standard error of the mean (SEM) unless otherwise indicated as standard deviation (SD).

## Data Availability

Generated raw data and/or analyzed data from the current study are available from the corresponding author on reasonable request.
